# Special Issue: Marine-Derived Compounds Applied in Cardiovascular Disease

**DOI:** 10.3390/md23120462

**Published:** 2025-11-29

**Authors:** Alexandros Tsoupras

**Affiliations:** Hephaestus Laboratory, School of Chemistry, Faculty of Sciences, Democritus University of Thrace, Kavala University Campus, 65404 Kavala, Greece; atsoupras@chem.duth.gr

Cardiovascular diseases (CVDs) are the leading cause of death worldwide. Several modifiable and non-modifiable risk factors initiate unfavorable processes linked to chronic inflammation, atherosclerosis, atherothrombosis, and CVD [1]. Although current preventive and therapeutic approaches are often effective, they are frequently associated with high financial costs, adverse side effects, and sustainability concerns.

This reality has intensified the demand for innovative strategies centered on natural bioactives derived from sustainable sources, and especially marine sources, which offer a promising alternative for the development of natural marine products with health-promoting properties against these disorders, including functional foods, nutraceuticals, and novel drug candidates. Marine sources and by-products of the marine industry are rich in bioactive compounds with strong cardio-protective properties [2,3]. Several natural products from marine sources have been studied and utilized for the development of nutraceutical supplements and drugs against inflammation-related chronic disorders, including atherosclerosis and CVD [4].

The evaluation of the preventative and/or therapeutic benefits of marine-derived compounds against cardiovascular diseases is of great importance. These compounds are particularly renowned for their preventative properties against the early stages of inflammation-related endothelial dysfunction; the recruitment and activation of platelets, monocytes and other leukocytes’ subtypes; the subsequent formation of atherosclerotic plaque; and their therapeutic effects during the final stages of the disease, including plaque rupture, atherothrombosis, and major cardiac events [2,3,4].

This Special Issue, “Marine-Derived Compounds Applied in Cardiovascular Dis-ease,” highlights the importance of bioactive compounds derived from several marine sources and their related by-products as inhibitors/modulators of these mechanisms with regard to cardioprotection and therapy. Research describing the isolation, identification and structural elucidation of marine-derived bioactive compounds for the prevention and therapy of atherosclerosis and CVD were also included. In addition, research on pharmacological characterization and Omics related to bioactivities, drug discovery, and drug development, and based on marine-derived bioactive compounds, including formulations and clinical trials, were also welcomed, along with relative reviews and meta-analyses.

*Contribution 1:* https://doi.org/10.3390/md22040187

The study of Rebeca André et al. concerns a purified aqueous extract of the brown alga/seaweed *Fucus vesiculosus*, rich in phlorotannins and peptides, that has previously been reported to inhibit cholesterol biosynthesis and intestinal absorption, and they authors examined how this extract affects metabolites and proteins in intestinal cells to better understand its mechanism of action with respect to lipid metabolism, particularly regarding the uptake and transport of dietary cholesterol [5]. A differentiated human colon adenocarcinoma cell line of Caco-2 enterocyte-like cells was treated with the extract and analyzed using untargeted metabolomics and proteomics. Metabolomic profiling revealed significant differences in glutathione levels between treated and control cells, along with increased levels of fatty acid amides in treated cells. Proteomic analysis showed a higher expression of phosphatidylinositol-3-phosphate 5-kinase (FAB1) and Niemann-Pick C1 protein (NPC1)—proteins associated with lipid metabolism and transport—in extract-treated cells compared with controls. Overall, in this study, both untargeted metabolomics and proteomics were employed to investigate the effects of *F. vesiculosus* on differentiated Caco-2 cells, providing new insights into the molecular actions of the extract’s components on intestinal cells.

*Contribution 2:* https://doi.org/10.3390/md21030136

Amandyne Linares-Maurizi et al. studied oxylipins from microalgae [6]. Microalgae are a rich source of bioactives, and especially of omega-3 and omega-6 polyunsaturated fatty acids (PUFAs), which, when undergoing oxidative degradation through radical or enzymatic processes, give rise to oxylipins—compounds recognized for their diverse bioactive properties. Amandyne Linares-Maurizi et al. sought to profile oxylipins from five microalgae species cultured under optimal conditions in 10 L photobioreactors. Cells were harvested during the exponential growth phase, extracted, and analyzed using LC-MS/MS to determine both qualitative and quantitative oxylipin profiles for each species. The five selected microalgae exhibited remarkable metabolic diversity, producing up to 33 non-enzymatic and 24 enzymatic oxylipins at varying concentrations. Collectively, these results underscore the potential of marine microalgae as valuable sources of bioactive lipid mediators, which may play important roles in preventive health, including modulation of inflammatory processes. The rich oxylipin mixtures produced by these species may be beneficial to biological systems and human health, providing, for example, antioxidant, anti-inflammatory, neuroprotective, immunomodulatory, and especially cardiovascular benefits.

*Contribution 3:* https://doi.org/10.3390/md20080515

Gabriele Carullo et al. worked on novel labdane diterpenes-based synthetic derivatives as vasodilators through specific biochemical signaling [7]. Sesquiterpenes such as leucodin and the labdane-type diterpene manool are natural compounds known for their strong in vitro vasorelaxant properties and in vivo hypotensive effects. Due to its structural resemblance to the sesquiterpene lactone (+)-sclareolide, this molecule was chosen as a scaffold for designing new vasoactive agents. A combination of functional assays, electrophysiological analyses, and molecular dynamics studies was conducted. Opening the five-membered lactone ring of (+)-sclareolide yielded a series of labdane-based small molecules that produced marked vasorelaxant effects in vitro. Electrophysiological experiments identified specific compounds as both CaV1.2 channel blockers and KCa1.1 channel activators, which were also confirmed in intact vascular tissue, probably due to their interaction with the dihydropyridine binding site. Docking and molecular dynamics simulations further clarified the molecular mechanisms underlying the compound’s inhibition of CaV1.2 channels and the activation of KCa1.1 channels. Additionally, these compounds also lowered coronary perfusion pressure and heart rate while prolonging atrioventricular node conduction and refractoriness, likely due to its Ca2+ antagonistic effects. Overall, these results indicate that the labdane scaffold is a promising foundation for developing new vasorelaxant agents with negative chronotropic properties that target the key pathways implicated in hypertension and ischemic cardiomyopathy.

*Contribution 4:* https://doi.org/10.3390/md22120554

Cholidis et al. reviewed the lipid composition of numerous shrimp species and examined the factors that can influence the lipid content of these crustaceans, along with their pleiotropic bioactivities and health-promoting properties, including their cardio-protective potential [3]. Particular attention was paid to the strong anti-inflammatory, antioxidant, and antithrombotic properties of shrimp-derived bioactive compounds, along with their potential roles in other conditions such as cancer, diabetes, and neurodegenerative diseases. The study also highlights the various health benefits associated with consuming shrimp lipid bioactives and using products enriched with shrimp lipid extracts, focusing on their mechanisms of action and the ways in which they interact with key biochemical pathways. Despite these promising findings in shrimp derived bioactives, additional research on this cultivable edible species is needed, especially considering current limitations and future opportunities, which were discussed in this work. Moreover, this paper induced a specific research group to further work on shrimp lipid bioactives for cardioprotection, with specific research being undertaken concerning conventional extracts rich in amphiphilic bioactives from shrimps with antioxidant and anti-inflammatory cardioprotective properties [8], as well as green extracts of amphiphilic bioactives from shrimps and their by-products, with similar benefits (data not yet published).

*Contribution 5:* https://doi.org/10.3390/md22040140

In the work of Mari Johannessen Walquist et al., marine-derived peptides with anti-hypertensive properties were reviewed, along with their prospects as ingredients for pharmaceuticals, supplements, and functional food products [9]. Hypertension is a major global health issue associated with cardiovascular disease and early mortality, driving the search for alternative therapies that avoid the side effects of current medications. The sustainable harvesting of low-trophic marine organisms not only supports food security but also provides access to diverse bioactive compounds, including peptides. Although they represent only a small portion of known natural products, peptides are highly suitable drug candidates due to their favorable size, structural stability, and resistance to degradation. The authors examined the antihypertensive potential of peptides and proteins derived from selected marine invertebrate phyla, discussing the methodologies used to study them and their relevance to pharmaceuticals, supplements, and functional foods. While substantial research has investigated the antihypertensive effects of certain marine invertebrates, many species remain unexplored. The wide range of assessment techniques—particularly for ACE (Angiotensin-Converting Enzyme) inhibition—pose challenges to comparing findings across studies. Furthermore, the predominance of in vitro and animal studies highlights the need for more clinical research to advance peptide-based candidates toward pharmaceutical application. Overall, in this review, a foundation for further investigation of these promising marine invertebrates was provided, which underscores the importance of balancing scientific progress with marine conservation to ensure sustainable resource use.

*Contribution 6:* https://doi.org/10.3390/md21030193

Finally, Wasim Akram et al. provided an excellent review of the marine-derived compounds applied in cardiovascular diseases [4]. The authors emphasized the richness of marine environments as reservoirs of novel bioactive metabolites with diverse pharmacological activities. Compounds derived from marine sources, such as omega-3 acid ethyl esters, xyloketal B, asperlin, and saringosterol, have shown promising effects across multiple CVDs. In the same article, the cardioprotective potential of marine-derived compounds in conditions such as hypertension, ischemic heart disease, myocardial infarction, and atherosclerosis were also highlighted. In addition, their current therapeutic applications, future prospects, and existing limitations were also examined.

Collectively, the contributions to this Special Issue highlight the multifaceted trajectory of research on natural marine bioactives for cardioprotection and CVD therapeutics. Several trends have emerged, as follows:

1. The new avenues are open through which future research may explore the therapeutic potential of macroalgae extracts in regulating lipid/cholesterol metabolism and developing functional foods or nutraceuticals aimed at cardiovascular health.
2. There is potential for future research to harness microalgae-derived lipid bioactives, and especially oxylipins, in the development of functional foods, nutraceuticals, and therapeutic agents aimed at preventing or managing inflammation-related diseases and promoting cardiovascular and overall health
3. We note the importance of the chemical modification of natural marine bioactives for the synthesis of innovative products for protection against CVD, as shown in the paradigm of optimized labdane-based derivatives as novel vasorelaxant agents, with potential therapeutic applications in hypertension, ischemic cardiomyopathy, and other cardiovascular disorders.
4. There is significant potential for future research to further explore shrimp-derived lipid bioactives as cardioprotective agents, including the development of both conventional and green extraction methods to harness their antioxidant, anti-inflammatory, and overall health-promoting properties.
5. There are future opportunities for valorizing marine-derived peptides as antihypertensive agents, including their clinical evaluation and development into pharmaceuticals, supplements, and functional foods, while ensuring the sustainable use of marine resources.
6. There is significant potential for future research to further explore marine-derived compounds for the development of novel cardioprotective therapies, with applications in hypertension, ischemic heart disease, myocardial infarction, and atherosclerosis, while not neglecting their current limitations.

The convergence of these strategies not only addresses challenges related to mitigating inflammation and CVD but also aligns with consumer demand for sustainable, safe, and effective natural marine products for cardioprotection.

Overall, this Special Issue illustrates the significant potential of natural marine bioactives for cardioprotection and therapeutic applications. Contributions concern a range of marine sources and bioactive classes, while collectively, these studies demonstrate the abilities of marine bioactives to modulate lipid and cholesterol metabolism, inhibit inflammatory processes, induce vasorelaxation, and provide antioxidant, anti-inflammatory, and cardioprotective effects. Furthermore, this Special Issue emphasizes the importance of sustainable harvesting, the chemical optimization of natural compounds, and translational research on functional foods, nutraceuticals, and pharmaceuticals. The contributions also identify key future research directions, such as the clinical evaluation of marine-derived peptides, the development of green extraction methods for shrimp bioactives, the exploration of macro- and microalgae metabolites for lipid regulation, and the design of novel vasorelaxant derivatives. Together, the articles contained herein underscore the promise of marine-derived compounds as safe, sustainable, and effective strategies for the prevention and treatment of CVD, while paving the way for innovative applications in human health.
